# Aquatic copper-containing nitrite reductase gene (*nirK*) phylogeny and environmental distribution

**DOI:** 10.3389/fmicb.2025.1635656

**Published:** 2025-09-17

**Authors:** Naomi Intrator, Bess B. Ward

**Affiliations:** Department of Geosciences, Princeton University, Princeton, NJ, United States

**Keywords:** nitrite reduction, *nirK*, oxygen minimum zone, nitrogen cycling, denitrification, AOA, ammonia oxidizing archaea, nitrification

## Abstract

Nitrite reduction is an essential step in the oceanic Nitrogen cycle. Nitrite reductase genes, mainly *nirS* and *nirK*, are found in dozens of phyla, are often associated with denitrifiers, ammonia- and nitrite-oxidizing bacteria (AOB and NOB) as well as ammonia-oxidizing archaea (AOA). *nirK* is found throughout the ocean, including in oxygenated surface water as well as in oxygen minimum zones (OMZs). The diverse and complex evolutionary history of the *nirK* genes makes it challenging to study the population structure and distribution of *nirK* containing organisms in the environment. The organisms containing *nirK* play key roles in the global nitrogen cycle, including the loss of fixed N, and have the potential to influence nitrous oxide (N_2_O) emissions via multiple pathways. This study surveyed the phylogeny and environmental distribution of over 12,000 *nirK* genes, focusing on those originating from marine and aquatic sources. Sequences were clustered into OTUs based on DNA sequence identity and their phylogeny and environmental sources were examined. The distribution of the sequences showed habitat separation within taxonomic groups, i.e., the majority of the OTUs were associated with only one environmental source. Bacterial *nirK* is more diverse phylogenetically and has a wider distribution across environmental sources than archaeal *nirK*. Most of the bacterial sequences were obtained from marine sediments, but there was variation in the dominant environmental source across phyla and classes. Archaeal sequences demonstrated niche separation between phyla as sequences from the more phylogenetically diverse phylum, Euryarchaeota, were all isolated from hypersaline environments while Nitrososphaerota sequences came from a wider range of environmental sources. This study expands the known diversity of *nirK* genes and provides a clearer picture of how *nirK* organisms are distributed across diverse environments.

## 1 Introduction

Nitrite (NO₂−) is an important source of biologically available fixed nitrogen—nitrogen that has been converted from atmospheric N₂ into usable forms—and a key intermediate in the global nitrogen cycle. In denitrification, the major route of fixed nitrogen loss from the biosphere to the atmosphere, nitrite is reduced to gaseous nitric oxide (NO) by one of two distinct nitrite reductases: the heme-coordinating cytochrome *cd_1_* type encoded by *nirS* genes and the copper-containing type encoded by *nirK* genes (Hochstein and Tomlinson, 1988; Glockner et al., 1993; Zumft, 1997). The complete pathway of denitrification, in which fixed nitrogen is successively reduced through anaerobic respiration, is as follows: nitrate (NO_3_^−^), NO_2_^−^, nitric oxide (NO), N_2_O, dinitrogen gas (N_2_) (Zumft, 1997). The intermediate N_2_O is a potent greenhouse gas, with approximately 300-fold greater global warming potential per molecule compared to carbon dioxide on the 100-year timescale, and is a significant agent in ozone depletion (Felgate et al., 2012; Ravishankara et al., 2009). Co-occurrence patterns of denitrification genes in cultivated microbes showed *nosZ* (the gene encoding the enzyme that reduces N_2_O) co-occurred more frequently with *nirS* than with *nirK* (Graf et al., 2014). This suggested that *nirS-*containing denitrifiers are more likely to perform complete denitrification and, therefore contribute less to N_2_O emissions (Graf et al., 2014), compared to those containing *nirK* (Hallin et al., 2018; Pold et al., 2024). A positive correlation between N_2_O emissions and *nirK* abundances, and qPCR analyses, revealed that only 10–30% of the *nirK-*containing denitrifiers also possessed *nosZ* (Clark et al., 2012). Therefore, the distribution and prevalence of *nirK* may influence net N_2_O emissions. Most previous research examining *nirK* focused on terrestrial sources, predominately soils, leaving *nirK* understudied in marine and other aquatic environments including hydrothermal vent systems, salt marshes, wastewaters, freshwater and estuary systems. Additionally, one-third of atmospheric N_2_O originates in aquatic environments, predominantly from microbial metabolism (Ciais et al., 2013), and ~20% of global N_2_O emissions are estimated to come from natural processes in the global ocean (Gluschankoff et al., 2023). Therefore, this study focuses on *nirK* from those marine and aquatic environmental sources.

In addition to the denitrifiers, nitrifying organisms (ammonia- and nitrite-oxidizing bacteria: AOB and NOB), also contain *nirK* (Cantera and Stein, 2007). AOB obtain energy from the oxidation of ammonia (NH_3_) to NO_2_^−^, while NOB gain energy from the oxidation of NO_2_^−^ to NO_3_^−^; therefore, each plays a significant role in the global nitrogen cycle (Prosser, 1986). In AOB *nirK* is involved in the production of N_2_O through ‘nitrifier denitrification’ under low oxygen conditions (Dundee and Hopkins, 2001; Shaw et al., 2005) and by other, somewhat undefined, pathways under oxic conditions (Freing et al., 2012). Nitrification has been found to contribute as much, or more, N_2_O to the atmosphere as heterotrophic denitrification in marine ecosystems (Dore et al., 1998; Ji et al., 2018; Buitenhuis et al., 2018). Lastly, *nirK* is also found in ammonia-oxidizing archaea (AOA) (Treusch et al., 2005); however, the function of *nirK* in AOA is still unclear. Both AOB and AOA also produce N_2_O under aerobic conditions, although the pathways are not clear and the involvement of *nirK* is not proven. N_2_O and N_2_O yield in AOB and AOA increase with increasing NO_2_^−^ concentrations and with decreasing oxygen levels (Santoro et al., 2011; Peng et al., 2015). However, culture experiments have shown AOA species were unable to produce N_2_O through the nitrifier denitrification pathway (Stieglmeier et al., 2014). A model utilizing archaeal *nirK* abundance could predict N_2_O production in oceanic oxygen minimum zones (OMZs), although not through the nitrifier denitrification pathway (Trimmer et al., 2016). A ‘hybrid formation’ of N₂O has been proposed in which NO, produced by the enzyme encoded by *nirK*, reacts with hydroxylamine, an intermediate of ammonia oxidation (Vajrala et al., 2012; Wan et al., 2023), to produce N₂O (Stieglmeier et al., 2014; Wan et al., 2023).

Although *nirK* has been known and studied in cultivated organisms for decades (Zumft, 1997), much is still unknown about the taxonomy, function, distribution, and biogeochemical impacts of *nirK*-containing organisms in the environment. This knowledge gap is partly attributed to the high taxonomic diversity of *nirK* that has been uncovered in the environment through PCR and sequence analyses (Helen et al., 2016; Liu et al., 2003; Oakley et al., 2006; Han et al., 2022; Zheng et al., 2024). Ongoing studies continue to expand our understanding of *nirK* diversity, suggesting that novel sequences are likely yet to be discovered. The large *nirK* sequence divergence observed may be due to *nirK*’s complex evolutionary history (Helen et al., 2016). Due to the polyphyletic distribution of *nirK* and denitrification gene phylogenies being incongruent with 16S rRNA, horizontal gene transfer has been proposed to have a substantial role in its evolution (Heylen et al., 2006; Jang et al., 2019). However, analysis of G + C and codon usage showed no evidence for horizontal gene transfer and therefore other phenomena, such as gene divergence/duplication and lineage sorting, may better explain *nirK*’s evolutionary history (Jones et al., 2008). Recent work by Ming et al. (2024) found *nirK*-carrying MAGs were more taxonomically diverse and less phylogenetically cohesive than *nirS*-carrying MAGs. They suggested that the scattered taxonomic distribution of *nirK*-containing organisms may reflect horizontal gene transfer and ecological flexibility. It has been hypothesized that sequence variation in *nirK* may be linked to ecological differences rather than ancestry alone (Pold et al., 2024).

We analyzed more than 12,000 *nirK* sequences from published databases, focusing on sequences from marine and other aquatic environments. *nirK-*containing organisms play a key role in fixed N loss pathways and in the production of N_2_O, both processes that may be linked to N_2_O emissions. Therefore, characterization of *nirK* gene diversity and environmental distribution will help to understand their biogeochemical impacts. This study analyzes the diversity and distribution of bacterial and archaeal *nirK* sequences focusing on the environmental sources (primarily marine and aquatic) of the gene to provide further insights into what environments and taxa are most influential in nitrogen transformation processes.

## 2 Materials and methods

### 2.1 Database compilation

To encompass the full diversity range of the *nirK* gene all gene features and CDS (coding sequences) of annotated *nirK* nucleotide sequences were downloaded from the National Center for Biotechnology Information (NCBI) Genbank database on June 4th, 2020. These *nirK* gene sequences were extracted from whole genomes as well as fragments from environmental clones and metagenomes. Duplicate sequences were then removed by keeping only the first instance of each sequence and its accession number, resulting in a total of 25,925 *nirK* sequences. Taxonomy was determined for each sequence using a modified code developed from the R package, Taxonomizr (Sherrill-Mix, 2018). Sequences were separated into domains: Bacteria, Archaea and Eukaryota (24,776, 971 and 208 sequences respectively). For this study Eukaryota sequences were removed from further analysis. Sequences were filtered by length and only sequences with length ≥400 bp for bacteria (11,638 sequences) and ≥300 bp (894 sequences) for archaea were retained for analysis.

### 2.2 Database processing

The 12,532 bacterial and archaeal *nirK* sequences were clustered using CD-HIT (Li and Godzik, 2006; Fu et al., 2012) with a defined threshold of 87% sequence identity (Taroncher-Oldenburg et al., 2003; Ward et al., 2007; Bulow et al., 2008). This threshold was chosen to cluster homologous genes at the level found to differentiate functional genes at the approximate level of species distinction initially reported for the *amoA* gene in AOB (Purkhold et al., 2000). The 87% threshold probably underestimates the number of “species” in the *nirK* database, compared to a genomic species definition of >95% Average Nucleotide Identity (ANI; Thompson et al., 2021). CD-HIT generated 2,185 bacterial and 86 archaeal clusters. The environmental sources of the sequences within each cluster were determined by searching NCBI and published literature. Metadata, including the environmental source and phylogenetic assignment (when available), was collected by searching the representative region using blastn optimized for highly similar sequences on NCBI (Camacho et al., 2009) (Supplementary Tables 1, 2).

Sequences were labeled with the following environmental sources: marine sediment, marine water column, marine OMZs, freshwater and estuary systems, hydrothermal vent systems, wastewater systems, aquatic other, terrestrial and animal (i.e., from the animal microbiome). Clusters that did not contain any marine or aquatic sequences were removed from further consideration. Approximately 30% of the 12,532 bacterial and archaeal *nirK* sequences were further analyzed after removing those outside the scope of this study, i.e., sequences that predominantly originated from terrestrial (mainly soil) sources and did not cluster with any aquatic sequences. The remaining clusters containing more than three sequences were each aligned with MAFFT v7.407 (Katoh and Standley, 2013), and a consensus sequence for each cluster was generated with emboss v6.6.0 (Stamatakis, 2014) (i.e., generating 180 bacterial and 19 archaeal consensus sequences). Clusters containing one or two sequences were removed from their cluster and treated as individual sequences that are identified by their accession numbers. This resulted in 180 marine and aquatic bacterial clusters (representing 2,327 sequences) and 655 unique bacterial sequences as well as 19 marine and aquatic archaeal clusters (representing 794 sequences) and 65 unique archaeal sequences.

Alignments of the consensus sequences and individual sequences for bacterial and archaeal sequences were made using MAFFT v7.407 (Katoh and Standley, 2013). Sequence lengths varied depending on how they were originally obtained (e.g., whole genomes vs. PCR fragments). To account for this, we extracted the longest continuous alignment region where the greatest number of clusters and individual sequences overlapped. This region was then used for downstream analyses. A 277 bp region was chosen for bacteria and a 161 bp region was used for archaea; however, 407 individual bacterial sequences did not align in the 277 bp region and were therefore not considered in further analyses (Supplementary Tables 1, 2). For the bacterial region, compared to the reference sequence of the *Nitrosomonas marina strain Nm71’s nirK* gene (1,105 bp: NZ_FOIA01000003.1), the 277-mer region is located around ~541–818 bp. The 161 bp archaeal region is around ~222–383 bp compared to the reference sequence of *Nitrosopumilus adriaticus strain NF*’s *nirK* gene (1,425 bp: CP011070.1, Bayer et al., 2016).

The probe finding algorithm of Bulow et al. (2008) was used to further group the 277 and 161 bp regions into operational taxonomic units (OTUs) and assign a representative sequence for each OTU. An identity threshold of 87% was used to provide the optimal discrimination between related sequences at approximately the species level for previously studied functional genes (Ward et al., 2007; Bulow et al., 2008). The algorithm produced 353 bacterial and 50 archaeal OTUs. Phylogenetic trees were generated with the aligned bacterial and archaeal OTUs using RAxML v8.2.12 (Stamatakis, 2014) and viewed and edited in iTOL v6 (Letunic and Bork, 2006). Ten bacterial clusters representing 40 sequences were removed from the phylogenetic tree (but can be found in Supplementary Table 1) because their extreme divergence prevented alignment. The phylogenetic trees represent a total of 2,552 bacterial sequences and 856 archaeal sequences.

## 3 Results

### 3.1 Aquatic *nirK* sequence compilation overview

**Figure 1:**
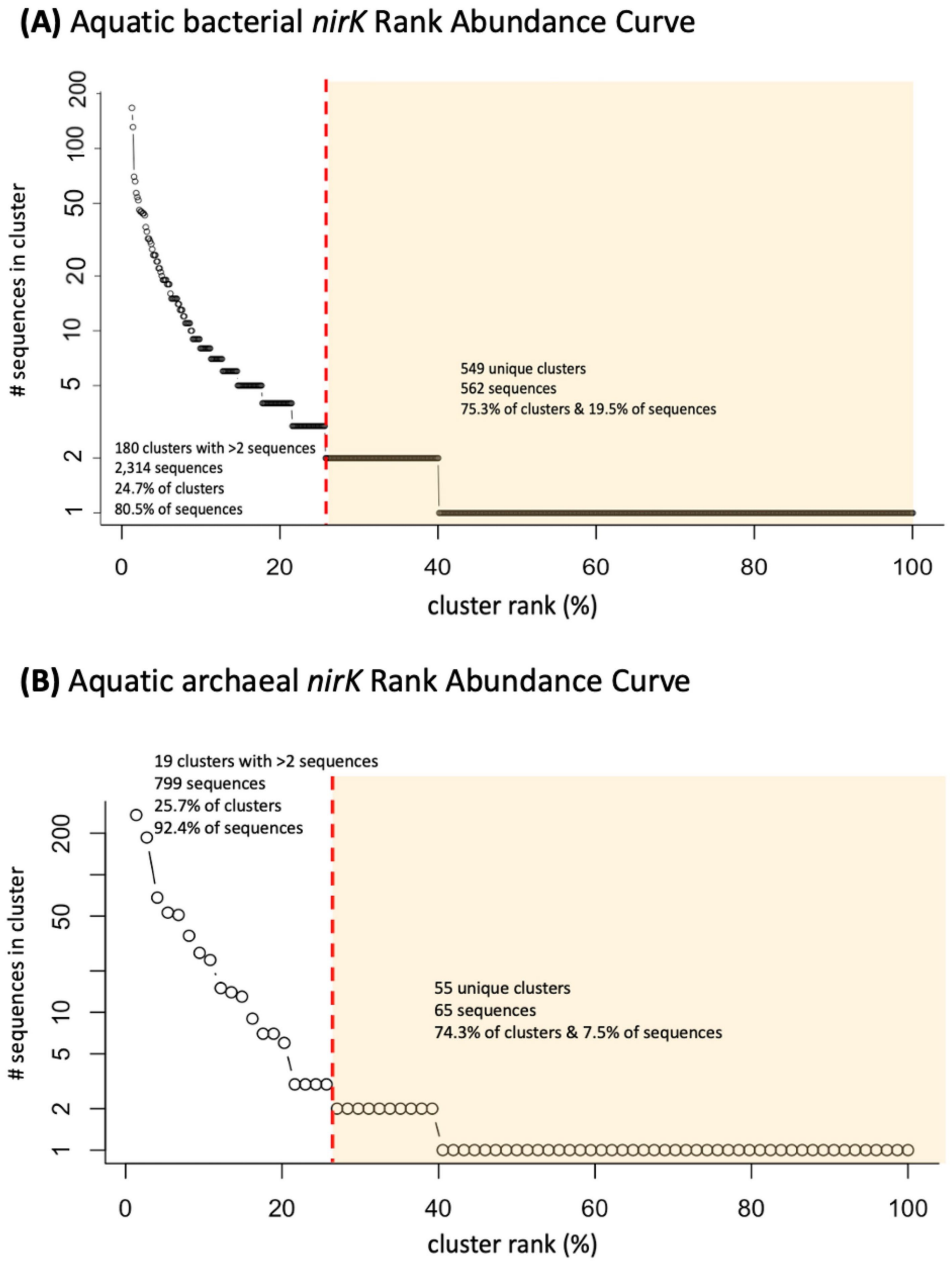
Rank abundance curve of analyzed clusters (circles) containing *nirK* sequences from marine or other aquatic environments. Distribution of **(A)** 729 bacterial and **(B)** 74 archaeal *nirK* clusters. Red dashed line separates clusters containing more than 2 sequences from those containing 2 or less, (unique/individual sequences) highlighted in yellow.

A total of 12,532 *nirK* sequences were compiled and processed. Following clustering using CD-HIT and removal of clusters comprised of only non-aquatic sequences, 2,876 unique aquatic bacterial and 864 archaeal *nirK* sequences were used for further analyses. Most of the sequences were derived from PCR amplification but several sequences were pulled from full genomes. These aquatic sequences grouped into 729 bacterial and 74 archaeal clusters. Of those, 180 bacterial and 19 archaeal clusters contained more than 2 sequences. These 180 bacterial and 19 archaeal aquatic clusters represent only 24.7 and 25.7% of the clusters, but account for 80.5% (2,314 sequences) and 92.4% (799 sequences) of the bacterial and archaeal sequences, respectively. The two largest aquatic clusters contain 167 bacterial and 271 archaeal sequences (5.8% of aquatic bacterial and 31.4% of archaeal sequences). The remaining bacterial and archaeal clusters, i.e., the unique individual sequences, represent 75.3 and 74.3% of the clusters respectively, and 19.5 and 7.5% of the bacterial and archaeal sequences (562 and 65 sequences respectively). This distribution of sequences shows that while the majority of the clusters are unique, most of the sequences group into larger clusters (Figure 1), particularly for archaeal *nirK* sequences.

### 3.2 Bacterial *nirK* environmental distribution and phylogeny

**Figure 2:**
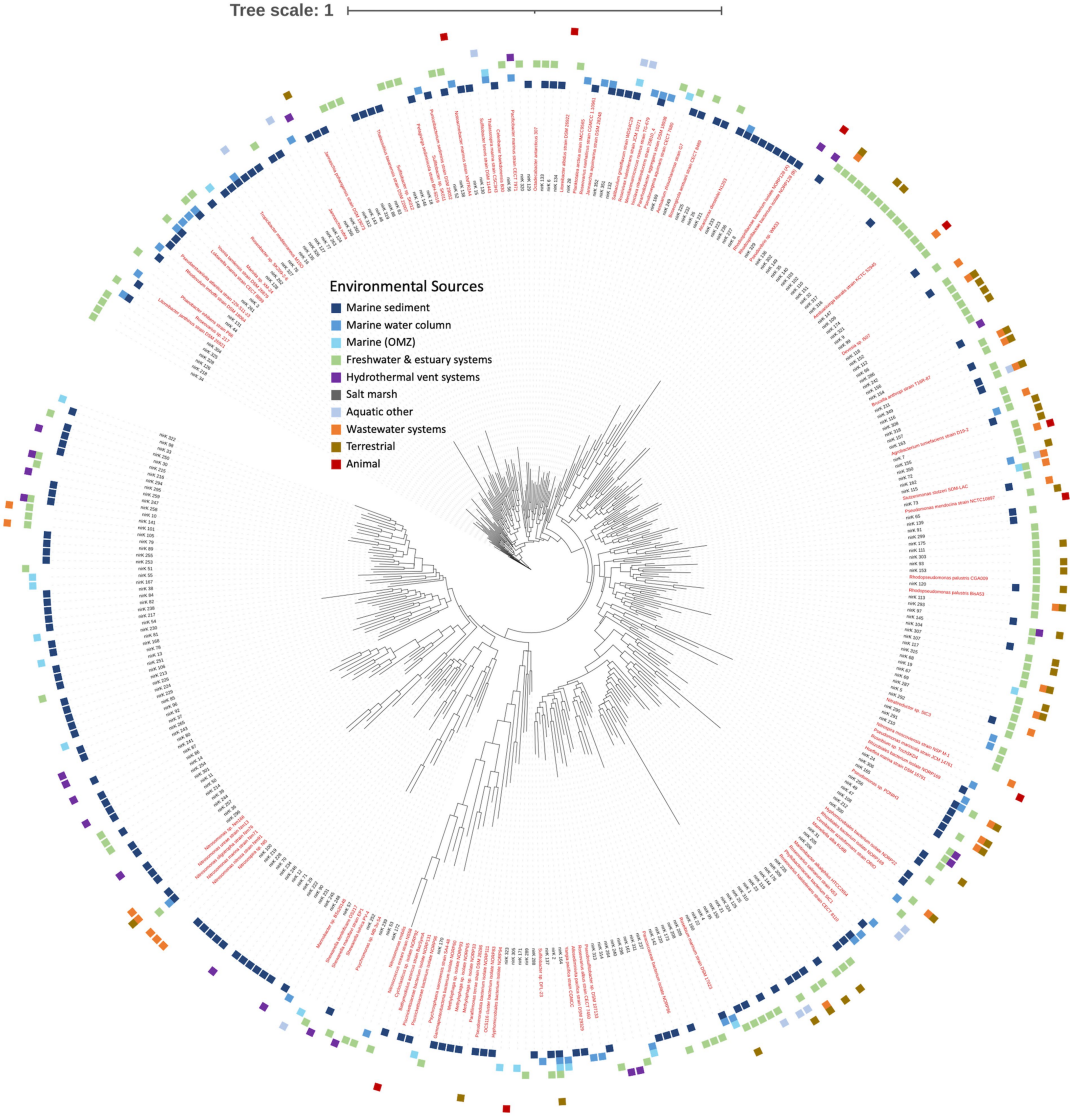
Phylogenetic tree of bacterial *nirK* based on OTU representative nucleotide sequences. Tree is based on 2,552 bacterial *nirK* sequences. Names of nodes written in red represent cultured/known organisms. Colored squares on the outermost rim represents the environmental sources of the sequences within each archetype (key in center).

**Figure 3:**
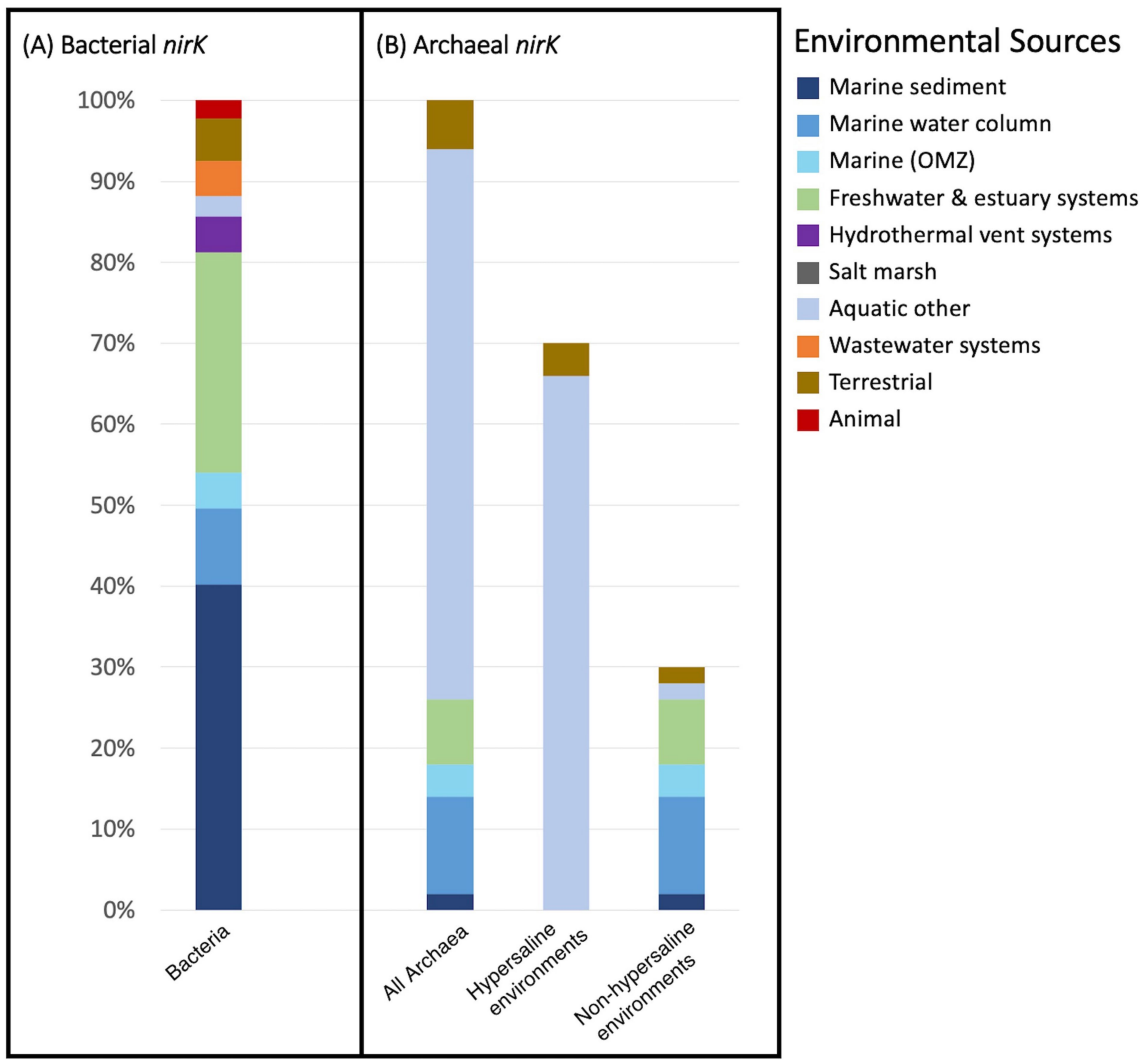
Percent of representative archetype sequences found from each environmental source. Bar plots display the percent of OTU representative sequences found from each environmental source for **(A)** the 353 bacterial OTUs and **(B)** the 58 archaeal OTUs. Archaeal OTUs are further separated into those obtained from hypersaline environments and non-hypersaline environments.

The majority of the *nirK* sequences were bacterial. A total of 2,552 bacterial *nirK* sequences were grouped into 353 OTUs (based on the aligned fragment, 277 bp), which were further examined. Two hundred fourteen OTUs represent 254 unique individual sequences only (i.e., do not contain clusters). The remaining 139 OTUs contained the majority of the sequences (2,298 sequences). The largest OTU represents 167 sequences (Figure 1A). Most of the OTUs (267 OTUs, 75.6%) contain sequences obtained from only one of the environmental sources. However, there are three OTUs (one uncultured Alphaproteobacterium and two unclassified) represented by sequences from five different environmental sources, and four OTUs (three unclassified and one *Pseudomonas* sp.) representing four different environmental sources. The remainder of the OTUs represent three or fewer different environmental sources. Figure 2 depicts the phylogeny of the bacterial *nirK* sequences based on the aligned 277 bp region of the gene and shows the spread of environmental sources across OTUs. The environmental sources for each OTU (based on the 277 bp OTU analysis) were identified and the total number of OTUs representing an environmental source was determined. Bacterial OTUs were predominantly obtained from marine sediment (40.2%) followed by freshwater and estuary systems (28%) (Figure 3A).

**Figure 4:**
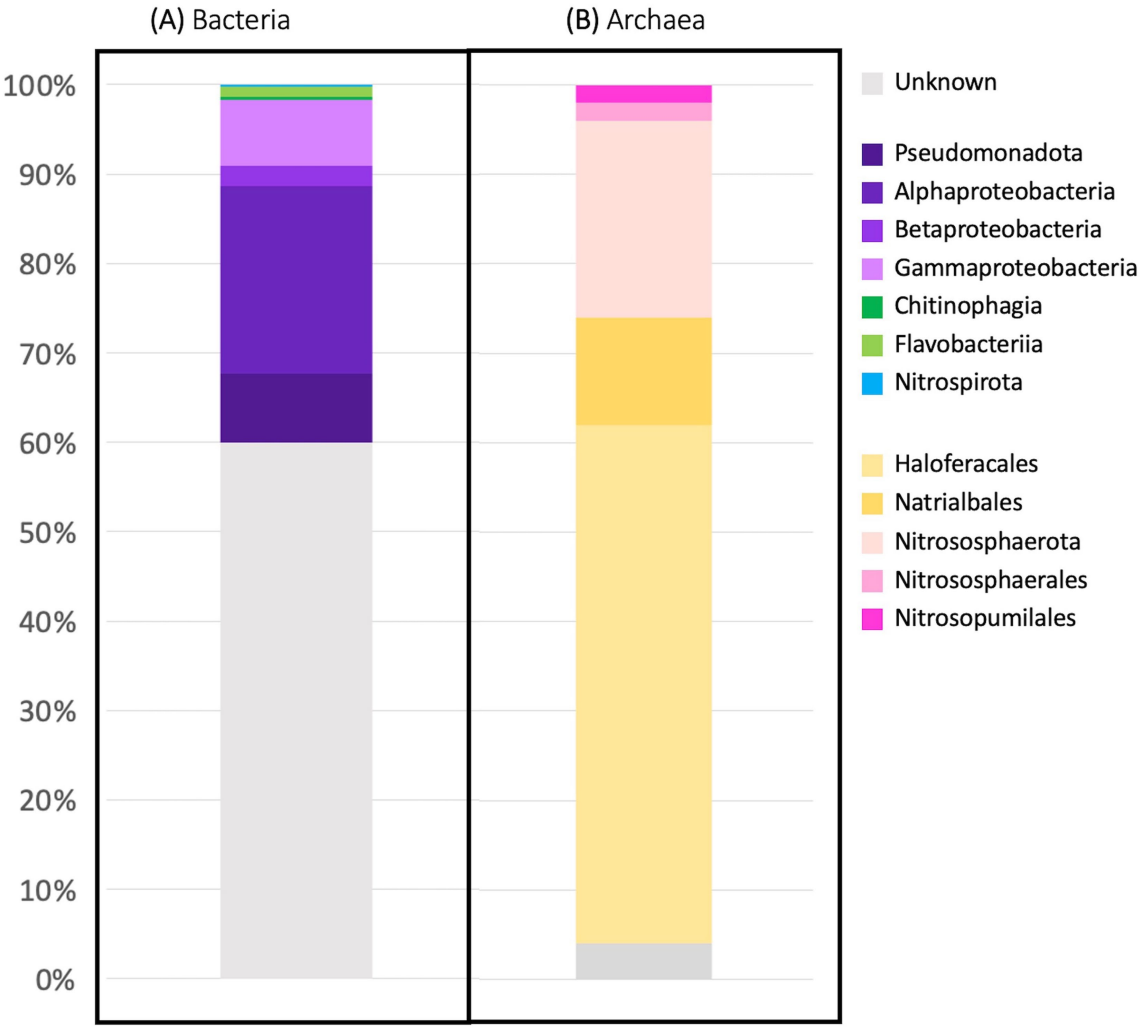
OTU taxonomy. Taxonomy of each OTU’s representative sequence was determined through phylogenetic inference for the **(A)** bacterial classes and **(B)** archaeal orders of *nirK* genes. Some Pseudomonadota bacterial genes could not be classified to the class level and are shown at the phylum level. *nirK* sequences that could not be classified below domain were characterized as unknown.

The taxonomy of each OTU’s representative sequence (Figure 4A) was determined through phylogenetic inference based on the tree in Figure 2. The phylum and class of the sequences within the bacterial *nirK* OTUs could be determined for 40 and 32.3% of OTUs respectively, while 60% of OTUs remained unclassified. Only 30.4, 28.2 and 18% could be assigned to the order, family, and genus, respectively, (Supplementary Table 1). The bacterial *nirK* genes that could be assigned phylogeny primarily consisted of sequences from the Pseudomonadota phylum (38.3%), with most classified as Alphaproteobacteria (20.9%), followed by Gammaproteobacteria (7.5%) and Betaproteobacteria (2.2%). The phylum Bacteroidota only made up 1.4% of OTUs and phylum Nitrospirota was the smallest represented phylum with only 1 OTU (0.3%). A few OTUs representing bacterial ammonia and nitrite oxidizers (AOB and NOB) were identified. Nine OTUs were classified as known AOB, encompassing a total of 15 sequences. Among these, most were classified as Betaproteobacteria (8 OTUs, 14 sequences), with only one OTU/sequence belonging to the Gammaproteobacteria class.

**Figure 5:**
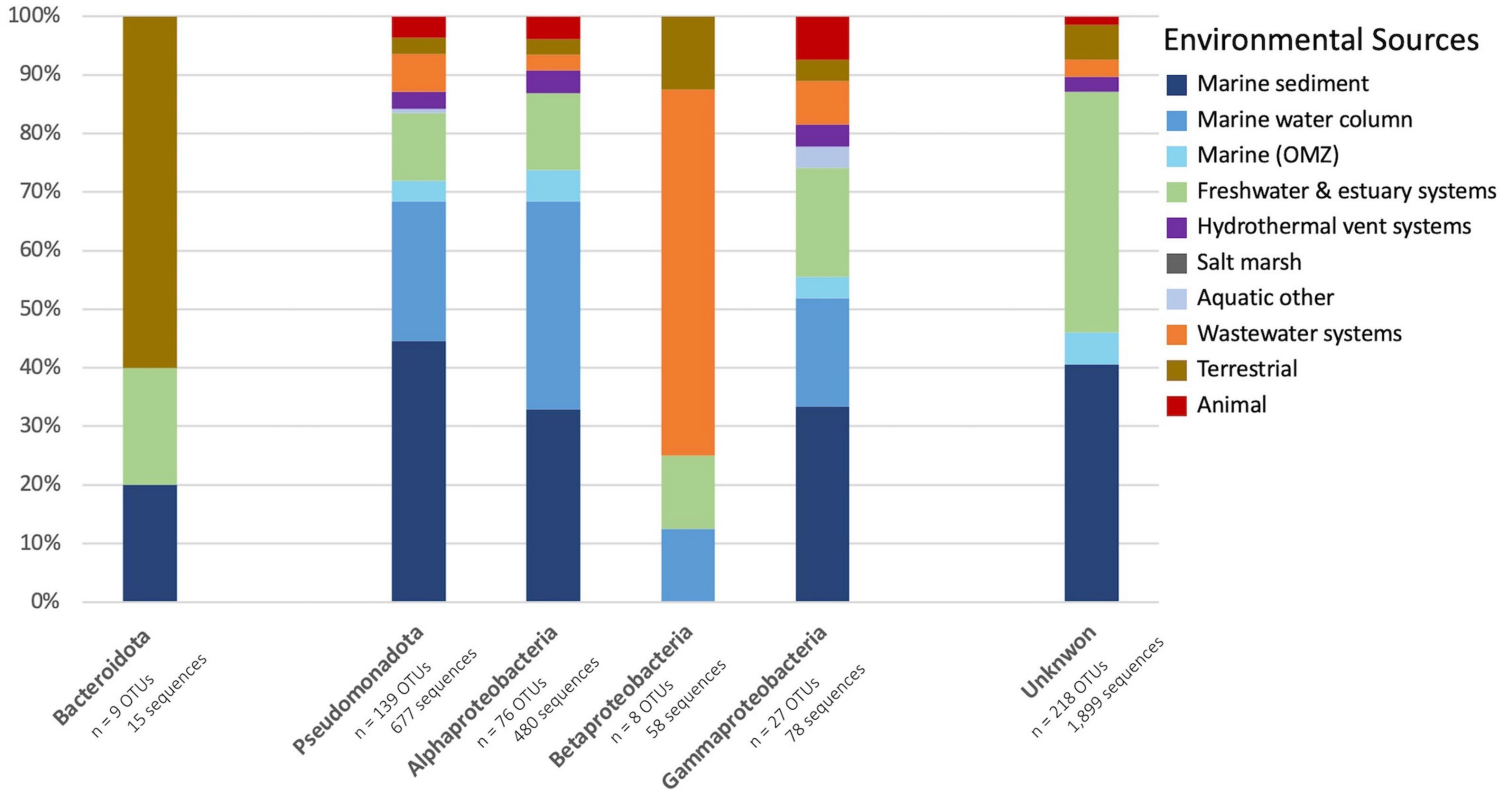
Percent of bacterial OTU from each environmental source. Bar plot displays the percent of OTU representative sequences obtained from each environmental source of the two bacterial phyla, Bacteroidota (left) and Pseudomonadota (middle; further broken down into classes Alphaproteobacteria, Betaproteobacteria, and Gammaproteobacteria), and the unknown sequences (right).

The environmental sources of the bacterial *nirK* sequences are shown in the outer wheel of Figure 2 while Figure 5 summarizes the breakdown of environmental sources within the phyla and those which could not be classified, i.e., unknown. The dominant phylum, Pseudomonadota, was examined further by determining what percent of OTUs came from each environmental source (Figure 5). Pseudomonadota, which represented 139 OTUs (677 sequences), was primarily obtained from marine sediments (44.6%), followed by the marine water column (23.7%). The Pseudomonadota classes were further analyzed. Alphaproteobacteria (76 OTUs, 480 sequences) was predominantly retrieved from the marine water column (35.5%), closely followed by marine sediment (32.9%). The main environmental source of Betaproteobacteria (8 OTUs, 58 sequences) was wastewater systems (62.5%). Lastly, Gammaproteobacteria sequences (27 OTUs, 78 sequences) were predominantly obtained from marine sediment (33.3%), followed by freshwater and estuarine systems (18.5%). On the other hand, the main environmental sources for Bacteroidota were terrestrial (60%); however, Bacteroidota only comprises 5 OTUs (15 sequences). The only OTU classified in the phylum Nitrospirota was obtained from a wastewater system. Most of the AOB OTUs were also obtained from wastewater systems. Those OTUs that could not be classified into phyla (218 OTUs, 1,899 sequences) were mostly obtained from marine sediment and freshwater and estuarine systems (both 38.1%), followed by hydrothermal vent systems (6.9%). No sequences with unknown taxonomy were obtained from the marine water column.

### 3.3 Archaeal *nirK* environmental distribution and phylogeny

**Figure 6:**
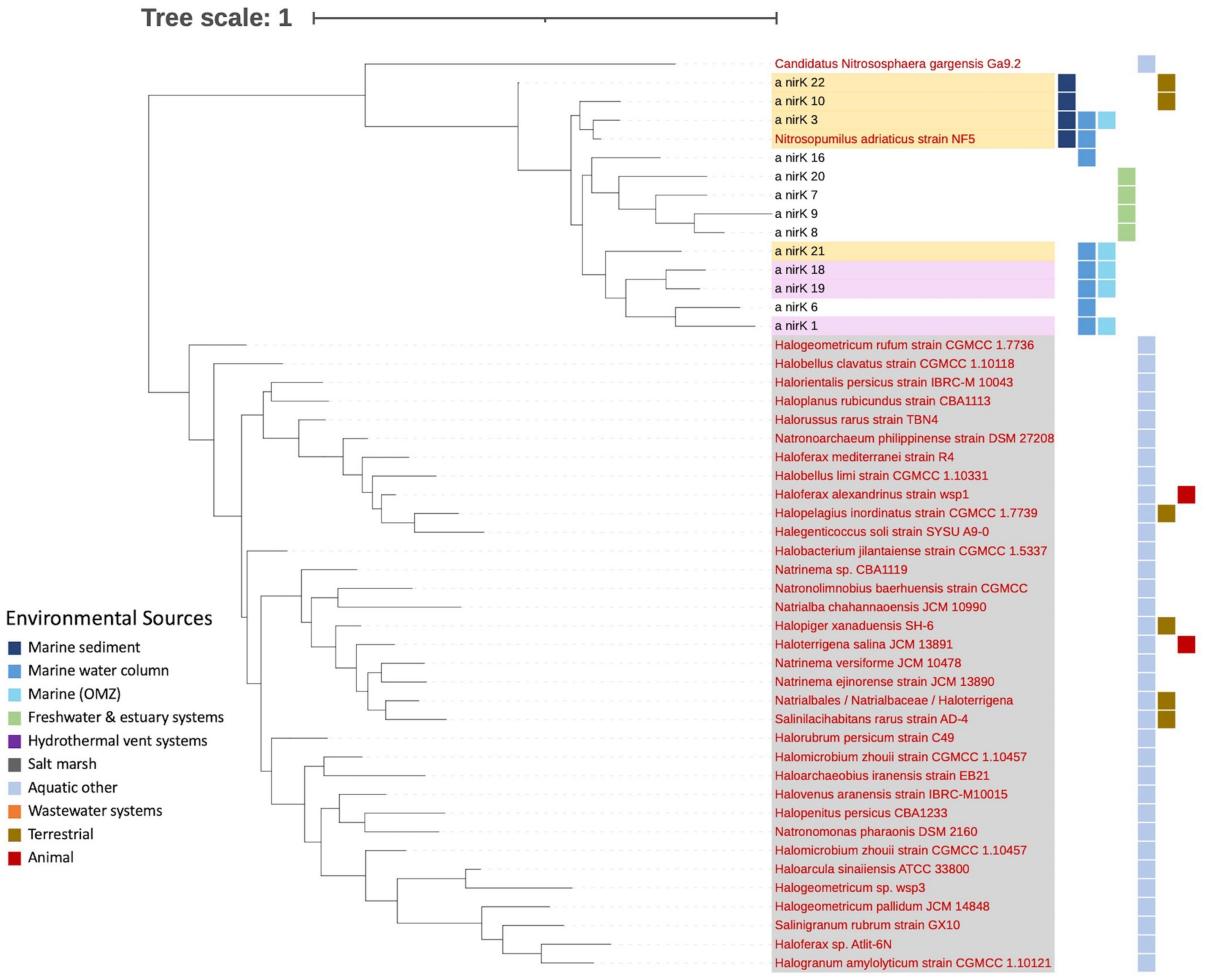
Phylogenetic tree of archaeal *nirK* based on OTU representative nucleotide sequences. Tree is based on 864 archaeal *nirK* sequences. Names of nodes written in red represent cultured/known organisms. Colored squares on the outermost rim represents the environmental sources of the sequences within each archetype (key on left). Hypersaline sequences are highlighted in gray. OTUs containing *AnirKa* and *AnirKb* from Lund et al. (2012) are highlighted in yellow and pink, respectively.

A total of 857 archaeal *nirK* sequences were grouped into 50 OTUs, which were further analyzed. Fifty-nine unique individual sequences made up 44 OTUs (containing 1–2 sequences each) while most of the sequences (805 sequences) were clustered into 14 OTUs. The largest archaeal OTU represented 275 sequences (Figure 1B). Similar to the bacterial *nirK* OTUs, most of the OTUs from the archaea (37 OTUs, 74%) represent only one environmental source. Only one OTU (260 sequences) represents sequences from three different environmental sources and was classified as an uncultured Nitrososphaerota (formerly known as Thaumarchaeota). The phylogenetic tree of the representative archaeal *nirK* OTU sequences, based on the 161 bp region of the gene (Figure 6) portrays the spread of the environmental sources across OTUs. Figure 3B depicts the fraction of environmental sources for all OTU representative sequences. The majority of the archaeal *nirK* sequences were obtained from “other” aquatic environments (68%) such as salt/saline lakes, solar salterns, and brines, i.e., mostly hypersaline environments. Hypersaline environments were separated from the remaining representative environmental sources (Figure 3B). Of the archaeal OTUs obtained from non-hypersaline environments, 40% were retrieved from the marine water column followed by 26.7% from freshwater and estuary systems.

Unlike the bacterial sequences, most of the archaeal *nirK* sequences could be classified into phylum/class (96%/74%) as well as 74% classified into order, family and genus (Figure 4B and Supplementary Table 2). Only two phyla were represented by the archaeal OTUs: Euryarchaeota (70%) and Nitrososphaerota (26%). All sequences originating from hypersaline environments were assigned to the phylum and class Euryarchaeota Halobacteria, while all those in non-hypersaline environments are within phylum Nitrososphaerota. Since Halobacteria within the phylum Euryarchaeota are exclusively found in hypersaline environments, the bar in Figure 3B labeled “hypersaline” reflects only Euryarchaeota, with environmental sources distributed as 97.1% aquatic (other) and 2.9% terrestrial. On the other hand, the bar showing non-hypersaline environments generally represents the environmental sources of the phylum Nitrososphaerota. The two unknown OTUs were obtained from freshwater and estuary systems.

## 4 Discussion

### 4.1 *nirK* diversity

Despite decades of research on the *nirK* gene, our knowledge of the function, distribution, and taxonomy of *nirK*-containing organisms in the environment remains incomplete. This is partly attributed to the broad taxonomic diversity of organisms harboring *nirK* genes, as identified through phylogenetic analysis and high-throughput sequencing of these genes in natural ecosystems (Cantera and Stein, 2007; Helen et al., 2016; Sun and Jiang, 2022). Differences in the structure of *nirK* genes, functional domains, operons, and transcriptional regulatory signals suggest multiple functions (Cantera and Stein, 2007). This extensive diversity is illustrated in Figure 1, where approximately 75.3% of bacterial clusters and 74.3% of archaeal clusters are unique sequences. After further grouping into OTUs, ~50% of both the bacterial and archaeal OTUs represent individual sequences, meaning approximately half of the branches on the phylogenetic trees (Figures 2, 6) represent unique sequences. Thus, although the OTU cutoff of 87% identity used here likely underestimates the number of different *nirK* sequences, the fact that the majority of the *nirK* sequences could not be assigned to phylogeny at the phylum level means that greater resolution in the “species” identity of the sequences would not be helpful at this time.

The high diversity of the *nirK* gene implies potential detection and analytical challenges, which suggests the size, distribution, and diversity of the microbial community of *nirK-*containing organisms is underestimated (Helen et al., 2016). A study by Cantera and Stein (2007) found previously published PCR primers used in various studies were unable to detect *nirK* genes from archaeal and several bacterial lineages from genomic DNA. In addition, Bonilla-Rosso et al. (2016) evaluated 79 *nirK* primers and determined there were no optimal universal primer pairs. Although this study examined a wide diversity of *nirK* sequences, we were limited to analyzing those publicly available from previous studies, and which met minimal length criteria, and therefore some diversity is likely missing due to the detection and analytical challenges recognized in environmental studies.

#### 4.1.1 Taxonomic diversity

The bacterial *nirK* represented the majority (95.5%) of the total aquatic *nirK* gene sequences analyzed here while archaeal sequences only made up 3.7%. These values are similar to a global prokaryotic census that found bacteria and archaea represented 93.3 and 6.7%, respectively, of over one billion prokaryotic reads (Louca et al., 2019). The smaller percentage of archaeal *nirK* (3.7% vs. 6.7%) compared to the global could be due to a research bias in microbial studies as more research has focused on bacteria.

The aquatic bacterial *nirK* sequences appear to be more phylogenetically diverse than the archaeal sequences (Figures 2, 6). The largest aquatic clusters contained 167 bacterial vs. 275 archaeal sequences, which represented 5.8% of aquatic bacterial and 32% archaeal sequences (Figure 1). Therefore, although more bacterial sequences were analyzed, they were more dissimilar to each other such that they grouped into smaller clusters. A higher diversity of bacterial vs. archaeal *nirK* sequences has been seen in other studies (Hansel et al., 2008; Helen et al., 2016; Ji et al., 2020) and is expected due to the significantly larger number of bacterial *nirK* sequences. In addition, a study by Hou et al. (2013), looking at *amoA,* which encodes the ammonia monooxygenase enzyme, and *nir*K/*nirS* genes, showed that despite a greater abundance of AOA vs. AOB, bacterial sequences still displayed greater phylogenetic diversity. In this study, the bacterial sequences came from a wider taxonomic range than observed for archaeal sequences (Figure 4). The bacterial *nirK* sequences in this study were identified as belonging to 3 distinct phyla, 6 classes and 53 genera, while archaeal sequences only included 2 phyla, 2 classes and 28 genera (Supplementary Tables 1, 2). Although they contain much unidentified and unexplored diversity within those phyla, the aquatic sequences analyzed here represent only a small fraction of the total global diversity of *nirK* described by Pold et al. (2024), who found *nirK* in at least 24 bacterial phyla, as well as the two archaeal phyla also represented here. Pold et al. (2024) divided the world into 28 biomes, only eight of which were the subject of the current study. Thus, a large portion of the bacterial *nirK* diversity found by Pold et al. (2024) came from terrestrial sources. Nevertheless, metagenomes from the eight aquatic biomes investigated by Pold et al. (2024), particularly the mid and high latitude ocean biomes, contained some of the highest *nirK* abundances and diversity per gigabase sequenced, which is consistent with the breadth of diversity we report here for aquatic systems.

Although Pseudomonadota was the largest phylum found among the bacterial sequences, most of the OTUs and sequences were unknown and could not be identified at the phylum level (60 and 73.3% respectively). Pseudomonadota (formerly known as the phylum Proteobacteria) has been identified as the dominant phylum in several environmental studies including those from marine sediments, marine OMZs, freshwater and estuary systems (respectively: Lee and Francis, 2017; Muck et al., 2019; Chen et al., 2017; Li et al., 2017). This is in contrast to the archaeal *nirK* sequences where the majority of the representative sequences could be identified and were classified in either the Nitrososphaerota or Euryarchaeota. Although the Euryarchaeota phylum represents the majority of the OTUs (70%), it only represents only a small portion of sequences 8.2% (i.e., the majority of the Euryarchaeota OTUs came from unique individual sequences). On the other hand, the Nitrososphaerota phylum represented 26% of OTUs but 87.2% of all archaeal sequences. This implies the Euryarchaeota phylum is more diverse than the Nitrososphaerota phylum in aquatic environments, which may be related to the environmental conditions in which these organisms are found (Castro-Fernandez et al., 2017; see section 4.2).

### 4.2 Ecological distribution (environmental sources) and niche differentiation

Due to the extensive and continually expanding understanding of *nirK* gene diversity, it is challenging to fully understand and study the ecological distribution of *nirK-*containing organisms. In this study we analyzed the environmental sources and environmental distribution of the *nirK* gene. Starting with a database of over 12,000 sequences, we clustered and identified *nirK* sequences through phylogenetic inference, subsequently identifying their environmental origins (Figures 2, 3, 6). Although our data compilation also included *nirK* sequences from published whole genomes, it is crucial to acknowledge that many sequences in this study were obtained via PCR amplification and clone libraries. Consequently, the patterns observed in the environmental sources and distribution discussed below are subject to PCR bias. With that caveat in mind, it is intriguing to explore the biogeographical and ecological patterns present in our extensive *nirK* database.

Most OTUs contained sequences from a single environmental source (75.6 and 74% of OTUs for bacterial and archaeal sequences), indicating they are unique to their specific environments (i.e., display biogeography). This supports previous findings of niche separation found between and within environmental sources (Oakley et al., 2006; Wang et al., 2014; Lee and Francis, 2017; Saarenheimo et al., 2017; Muck et al., 2019; Hatzenpichler, 2012 and references therein). In this study, bacterial OTUs have a wider distribution across our defined environmental sources than archaeal OTUs (Figures 2, 3, 6). This implies bacterial *nirK* microbes are adapted to a broader range of environments than archaeal *nirK* organisms, consistent with the greater gene diversity observed. Pold et al. (2024) found that *nirK* diversity and abundance usually far exceeded that of *nirS* (across 28 different environments), and that specific clades of both genes were associated with specific environments. *nirS* and *nirK* are associated with different biomes and different environmental conditions, suggesting different functionality.

Bacterial *nirK* OTUs were predominantly retrieved from marine sediment while the majority of the archaeal OTUs were obtained from other aquatic environments. These other aquatic environments are primarily extreme conditions, particularly those with high salinity (e.g., salt/saline lakes, solar salterns, and brines). Niche differentiation was evident, as all sequences originating from hypersaline environments were assigned to the Euryarchaeota phylum. The Euryarchaeota phylum exhibited greater phylogenetic diversity, containing more unique individual sequences than the other archaeal phylum found in our study, Nitrososphaerota. This diversity in Euryarchaeota is underpinned by their broad range of metabolic capabilities and environmental adaptations, allowing them to thrive in various extreme environments (Castro-Fernandez et al., 2017; Martinez-Espinosa, 2020). On the other hand, Nitrososphaerota, while being phylogenetically less diverse, exhibits greater ecological diversity as it is found in a wider range of environmental sources (Figure 3B). This broad ecological distribution reflects its critical role in the global nitrogen cycle, particularly in ammonia oxidation (i.e., AOA), which is a key component of the N cycle in all environments (Zheng et al., 2024). However, the functional role of *nirK* in these AOA remains uncertain (Lund et al., 2012; Kop et al., 2024). Thus, this niche differentiation implies archaeal *nirK*-containing organisms contribute to the nitrogen cycle in distinct ways: Euryarchaeota likely engaging in denitrification in hypersaline environments (Torregrosa-Crespo et al., 2018) and Nitrososphaerota in ammonia oxidation in a wider range of environments.

A similar study (Biller et al., 2012) focused on the archaeal *amoA* gene, which encodes the *α*-subunit of the ammonia monooxygenase enzyme required for ammonia oxidation. That study aimed to understand the ecological factors influencing the distribution and diversity of AOA. Examining over 8,000 *amoA* sequences, they found, similar to our study, that the majority of sequences tend to cluster with others from the same environment. The findings by Biller et al. (2012), like ours, support the existence of multiple distinct aquatic AOA clades in the environment. They suggested that factors such as salinity, depth, and temperature could be selective pressures driving the niche partitioning of AOA and influencing *amoA* diversity. Their study found ~75% of their total *amoA* sequences to be unique, very similar to the 74.3% we found for archaeal *nirK*. However, unlike our study, the majority of their *amoA* sequences came from coastal sediments, whereas our study found that the *nirK* sequences from Nitrososphaerota, likely representing AOA, were predominantly obtained from the marine water column. This discrepancy maybe due to a sampling bias between research conducted on *amoA* versus *nirK*. Additionally, 16S rRNA gene abundances of Thaumarchaeota (Nitrososphaerota) were greater than AOA *amoA* suggesting not all Thaumarchaeota contain *amoA* or were missed due to prior primer bias (Tang et al., 2023). Biller et al. (2012) also revealed, similar to our study, hydrothermal vents and wastewater systems were underrepresented environments in terms of total numbers of sequences (Figure 3B). Notably, 75% of the AOB OTUs were obtained from wastewater systems. This distribution is consistent with previous studies that have highlighted the prevalence of AOB and Betaproteobacteria in wastewater treatment systems (Yin et al., 2018; Wu et al., 2016) while AOA dominate marine systems.

The diversity of Nitrososphaerota (Thaumarchaeal) *nirK* genes in coastal and marine environments was previously explored by Lund et al. (2012), who identified two distinct groups, AnirKa and AnirKb, based on primer sets. The two variants showed contrasting distributions in the water column of Monterey Bay and the California Current. AnirKa was more prevalent in the epi- to mesopelagic Monterey Bay, while AnirKb was dominant in the meso- to bathypelagic California Current. In addition, only AnirKa could be detected in sediments. This separation persists in our analyses, as the AnirKa and AnirKb sequences of Lund et al. (2012) fell into separate clusters (Figure 6; yellow represents AnirKa, pink represents AnirKb). This separation further demonstrates niche differentiation between *nirK* sequences. Metadata such as depth, as shown by Lund et al. (2012), could reveal further unseen separations in the rest of our sequences and provide deeper insights into the ecological roles and adaptations of these Nitrososphaerota groups in different marine environments. *nirK* resolved the diversity of AOA better than *amoA*, distinguishing three pelagic ecotypes in the marine water column (Reij et al., 2019).

The widespread distribution and overall large diversity of *nirK*-containing organisms suggest that the ability to reduce nitrite is an ecologically significant and favorable trait. While not solely linked to canonical denitrification, this ability may partially regulate net N_2_O emissions (Hallin et al., 2018; Clark et al., 2012; Shaw et al., 2005). Such large gene diversity and distribution suggest differing *nirK-*containing organisms will have differing nitrite affinities and reduction efficiencies, potentially influenced by genetic diversity and ecological adaptations. Several factors such as oxygen concentration, nutrient availability, redox conditions, etc., could influence the observed environmental distribution and should be examined further. For instance, in a study in the Yellow River Estuary phylogenetic analysis revealed differing *nirK* clusters between sites of high versus low dissolved oxygen concentrations (Li et al., 2017). Principal-component analysis of biogeochemical data and denitrifying organisms conducted along the Pacific coast of Mexico revealed nitrate concentrations and oxygen levels were key factors controlling the structure of denitrifying communities (Liu et al., 2003). Similarly, in rice paddies changes in redox chemistry in the water led to shifts in the active microbial community and expression of functional genes including *nirK* (Yoshida et al., 2009). Phylogenetically distinct organisms exhibit unique ranges and tolerances to environmental conditions, often enabling them to occupy specific ecological niches. The spread of these diverse taxa across environments has implications or global N-cycling and is an active area of study.

Niche differentiation was also observed between and within the bacterial phyla, Pseudomonadota and Bacteroidota (Figure 5). Bacteroidota sequences were primarily obtained from terrestrial environments and Pseudomonadota from marine sediment. The Pseudomonadota classes were further analyzed to resolve finer scale patterns in environmental distribution. The largest class, Alphaproteobacteria, was mainly derived from the marine water column and marine sediment (35.5 and 32.9% respectively). Similarly, Gammaproteobacteria sequences were predominantly obtained from marine sediment (33.3%), followed by the marine water column and freshwater and estuary systems (both 18.5%). However, the smallest class, Betaproteobacteria, was dominated by sequences from wastewater systems (62.5%), including the AOB. The variation in environmental sources between classes suggests that environmental selection operates at a finer scale than just the phylum level. This indicates the need for further studies to determine the precise extent of biogeography and niche differentiation at these finer taxonomic levels. Niche partitioning has been found in relation to particulate concentrations, salinity, nitrogen concentrations, organic matter, temperature, pH, oxygen levels, etc. (Muck et al., 2019; Jones and Hallin, 2010; Lee and Francis, 2017; Hatzenpichler, 2012); likely, there is no single driving factor that controls *nirK* niche separation in the wider context of microbial genomes. Lastly, the majority of the bacterial *nirK* sequences could not be classified into specific phyla. These unknown sequences were predominantly obtained from marine sediment and freshwater and estuary systems. This indicates that these environments contain phylogenetic and metabolic diversity that should be explored to elucidate the potential roles for *nirK* in nitrogen cycle and respiratory functions.

### 4.3 Summary and future directions

This study expands our understanding of the diversity and ecological distribution of *nirK*-containing organisms in marine and other aquatic environments. The *nirK* gene is found in organisms in a wide range of genera inhabiting diverse environments. The organisms containing *nirK* play essential roles in the global nitrogen cycle through a variety of pathways and have the potential to influence N_2_O emissions. Although *nirK* was characterized over two decades ago (Zumft, 1997), much remains unknown about the distribution, taxonomy, and biogeochemical impacts on the environment. This study analyzed over 12,000 *nirK* sequences and found large diversity of the gene: based on clustering at 87% identity, the majority of the OTUs were unique. This extensive sequence diversity introduces detection and analytical challenges. PCR primers can only detect a limited range of the phylogenetically diverse *nirK* and therefore universal PCR primers for *nirK* are not possible. In turn, it is likely that *nirK* diversity and environmental abundance are greatly underestimated. The ecological significance of this underestimation should be further examined, preferably using metagenomics to avoid PCR primer bias.

We found bacterial *nirK* to have a wider taxonomic range and distribution across environmental sources compared to archaeal *nirK*. Differences in community composition and environmental distribution among *nirK*-containing organisms are likely to impact environmental nitrogen cycling causing various ecological and environmental consequences, because the function of *nirK* varies among organisms (e.g., denitrifiers, NOB, AOA, and AOB). However, the exploration of these consequences is beyond the scope of this study and additional field studies are required to understand the biogeochemical impacts of this variation. The majority of the OTUs analyzed in this study were each obtained from only one environmental source, suggesting most *nirK* organisms are specific to an environment. The spread of the diverse taxa across environments has implications for nitrogen cycling and its connections with other diverse metabolisms.

This study examined the environmental distribution of the *nirK* gene with a focus on marine and other aquatic environments to explore the breadth of environmental sources. The majority of the sequences were bacterial, which were predominately obtained from marine sediments. In addition, most bacterial OTUs could not be taxonomically classified even to the phylum level. Pseudomonadota was the largest identified phylum of bacterial *nirK*, most of which were Alphaproteobacteria. Niche differentiation was observed between and within the bacterial phyla. Bacteroidota sequences were primarily obtained from terrestrial environments and Pseudomonadota from marine sediment. Finer scale patterns in environmental distribution were observed by looking at the Pseudomonadota classes, where Alpha- and Gammaproteobacteria were primarily isolated from marine sources (water column and sediment), while Betaproteobacteria was dominated by sequences from wastewater systems. Archaeal sequences also demonstrated niche differentiation as the more phylogenetically diverse phylum, Euryarchaeota, were all isolated from extreme hypersaline environments while Nitrososphaerota sequences came from a wider range of environmental sources. The mechanisms responsible for the environmental distribution and niche separation of *nirK*, such as nutrient availability, oxygen concentration, redox state, etc., are not well understood and should be examined further as it impacts nitrogen and biogeochemical cycling.

A major challenge in establishing *nirK* sequence diversity in an environmental context was the lack of standardized environmental metadata such as temperature, salinity, latitude and longitude, dissolved oxygen concentrations, depth, etc. Understanding the factors that influence the environmental distribution and niche separation of *nirK* in different environments requires such data. These limitations highlight the necessity of additional studies on cultivated organisms to better understand the specific factors driving *nirK* niche differentiation.

## Data Availability

The original contributions presented in the study are included in the article/Supplementary material, further inquiries can be directed to the corresponding author/s.

## Supplementary material

SUPPLEMENTARY TABLE 1 Archetype sequence information and metadata for bacterial *nirK* OTUs.

SUPPLEMENTARY TABLE 2 Archetype sequence information and metadata for archaeal *nirK* OTUs.
